# Establishment of the Occlusion Plane Using Broadrick Occlusal Plane Analyzer for Immediate Complete Dentures

**DOI:** 10.3390/dj13120605

**Published:** 2025-12-16

**Authors:** Marisol López-Pulido, Luis Angel Sánchez-Chaidez, Kenji Miguel Ishino-Cortez, Verónica Manteca-López, Andréa Dolores Correia Miranda Valdivia

**Affiliations:** 1 Department of Prosthodontics, Faculty of Dentistry, Autonomous University of Guadalajara, Guadalajara C.P 44160, Jalisco, Mexico; marisollopez@edu.uag.mx (M.L.-P.); langel.sanchez@edu.uag.mx (L.A.S.-C.); veronica.manteca@edu.uag.mx (V.M.-L.); 2 Department of Periodontics, Faculty of Dentistry, Autonomous University of Guadalajara, Guadalajara C.P 44160, Jalisco, Mexico; kenji.ishino@edu.uag.mx; 3 Department of Specialized Dentistry, Faculty of Dentistry, Autonomous University of Guadalajara, Calle Escorza 526, Colonia Americana, Guadalajara C.P 44160, Jalisco, Mexico

**Keywords:** denture, complete, immediate, occlusal plane, balanced dental occlusions

## Abstract

**Background/Objectives**: Pathological tooth loss resulting from poor oral hygiene or systemic diseases can lead to partial edentulism, affecting patients both psychologically and physically. These consequences include facial height reduction, temporomandibular dysfunction, and impaired phonetics and mastication. Immediate complete dentures are often an effective provisional solution during the transition to full edentulism; however, establishing the occlusal plane can be challenging when remaining teeth prevent a conventional wax try-in. This clinical case aims to present a qualitative clinical case study of a single patient, illustrating the use of the Broadrick Occlusal Plane Analyzer (BOPA) for the establishment of an occlusal plane in harmony with the anterior and condylar guidance. **Methods**: A 51-year-old male patient presented to the Department of Prosthodontics at the School of Dentistry, Autonomous University of Guadalajara, with partial edentulism, periodontal disease, and generalized Grade III tooth mobility. Immediate maxillary and mandibular complete dentures were selected as the treatment of choice. Due to the presence of remaining teeth that hindered clinical determination of the occlusal plane, the BOPA was used during the denture design process. **Results**: Anatomical landmarks were combined with BOPA tracing to establish an occlusal plane harmonious with anterior and condylar guidance. The center of the curve was modified to accommodate anatomic variability in anteroposterior reference points. **Conclusions**: The use of the Broadrick Occlusal Plane Analyzer facilitated the accurate determination of the occlusal plane for the fabrication of immediate complete dentures in a patient where clinical assessment was limited. This modification allowed the establishment of a bilateral balanced occlusal scheme, contributing to functional and acceptable provisional oral rehabilitation during postoperative alveolar healing.

## 1. Introduction

The pathological loss of one or more teeth due to poor oral hygiene or systemic diseases that induce periodontal disease can cause partial edentulism, which generates physical changes such as a decrease in the lower third of the face, temporomandibular dysfunction, changes in phonetics, mastication, displacement of proximal contacts, food impaction, occlusal interference, and anterior overload [1,2]. To determine the appropriate dental treatment, the remaining teeth and their strategic position within the oral cavity are clinically evaluated. On many occasions, the selected abutments are scarce, and the available treatments are limited to removable partial dentures, dome-supported overdentures [1,2], or implant-supported prosthesis —this last option often being inaccessible due to the patient’s general health condition and financial limitations [3,4]. Given these circumstances, a transitional treatment is proposed, allowing the patient to move from having a few remaining teeth in poor condition to a state of complete edentulism, followed by the placement of a complete denture.

An immediate complete denture is a more affordable option, particularly for patients who are not candidates for implant treatment or cannot afford it, or when the remaining abutments are irreversibly compromised, necessitating the extraction of all teeth in one or both arches as part of the treatment plan [1,5]. Immediate dentures replace missing teeth as well as maxillary and mandibular structures and are placed immediately after the extraction of periodontally affected teeth. They may also act as a splint, aiding hemostasis following surgery by promoting wound healing, preventing trauma, preserving facial appearance and height, maintaining muscle tone, and improving phonetics [5,6]. The successful outcome of an immediate complete prosthesis depends on providing both physiological and psychological comfort to the patient, which is influenced by the quality of prosthesis fabrication, the morphological characteristics of the oral mucosa, and the establishment of a new functional occlusal pattern [7].

The basic concepts of occlusion are still applied today, as creating an incorrect occlusal scheme can affect the retention, stability, and support of a complete prosthesis. The bilateral balanced occlusal scheme is one of the most commonly used, as it allows for the proper equilibrium of the artificial teeth of the prosthesis during lateral and protrusive movements [7].

This is achieved by maintaining occlusal contacts not only in centric relation but also during functional mandibular movements, including lateral and protrusive excursions [7]. A key step in establishing this occlusal pattern, which should ideally be functional, is the determination of the occlusal plane, which should closely resemble the position previously occupied by the natural teeth in relation to the incisal and occlusal surfaces [7,8,9]. Various techniques are available to determine the occlusal plane, including the use of anatomical landmarks such as the tongue, retromolar pad, the labial commissure and Stensen’s duct; reference planes like the ala-tragus line or Camper’s plane are also commonly used [4,8,9,10]. Additionally, the placement of teeth in the prosthesis during clinical try-in serves as a guide [11,12]. In partial denture patients scheduled for immediate complete denture restoration following surgical extraction of the remaining teeth, clinical trial of the waxed-up teeth is often hampered by the presence of the remaining teeth, which may present a clinical challenge for the rehabilitation practitioner when designing the prosthesis. In these cases, alternative methods can be used to establish the occlusal plane, based on compensatory curves of the condylar path and mandibular movements [13]. The determination of the occlusal plane using the anterior–posterior curve (Spee) and the lateral curve (Wilson) relies on the alignment of the incisal edges and the occlusal surfaces of artificial teeth, which are designed to simulate an ideal natural dentition and achieve harmony between the anterior teeth and condylar guidance [11,13]. A key factor in this process is the accurate positioning of the central incisors; when these are properly placed, the occlusal surfaces of the remaining teeth are more likely to align correctly [14]. The “Broadrick Occlusal Plane Analyzer” (BOPA) is an instrument used to determine the most probable location of the center of the anterior–posterior curve after the upper and lower incisal edges have been properly aligned during mounting on the articulator. It helps to recreate the position of the posterior teeth in harmony with the anterior teeth and the condylar guidance. This harmony allows for the complete separation of the posterior teeth during mandibular protrusion [11]. While this separation may be considered a disadvantage in terms of prosthesis retention and stability [7], it is important to recognize that the center of the curve is not a fixed or constant reference [11,15]. For esthetic or functional reasons. The radius of the curve can be adjusted; this modification can create posterior occlusal contacts not only in centric relation but also during functional mandibular movements, taking into account these contacts characteristic of a bilateral balanced occlusion [7,11].

This clinical case is presented with a qualitative approach to illustrate and describe an option for establishing the occlusal plane during the fabrication of an immediate complete denture when the remaining teeth prevent a wax try-in. This is achieved through the use of the Broadrick Occlusal Plane Analyzer, modifying the curve’s center by selecting an anatomical point as a reference to achieve a bilateral balanced occlusion in a patient who maintained a reliable reference of the incisal edge.

## 2. Case Report

**Figure 1:**
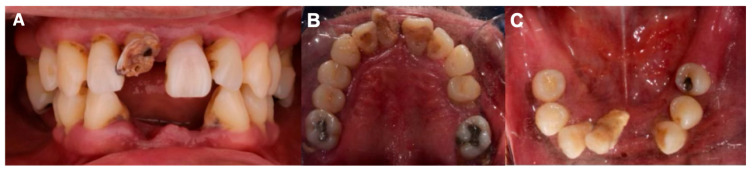
(**A**) Intraoral photograph showing the initial periodontal status. (**B**) Upper arch. (**C**) Lower arch.

A 51-year-old male patient arrived at the Prosthodontics Department of the Faculty of Dentistry, Autonomous University of Guadalajara, with the chief complaint: “I have lost several teeth and my gums hurt.” Intraoral examination revealed a partially edentulous condition, periodontal disease, and generalized grade III tooth mobility (Figure 1). Radiographic analysis showed extensive and severe bone loss, compromising the prognosis of the remaining teeth. Due to the unfavorable prognosis, multiple extractions were planned, and immediate complete dentures were chosen as a transitional treatment prior to the eventual placement of definitive complete dentures.

**Figure 2:**
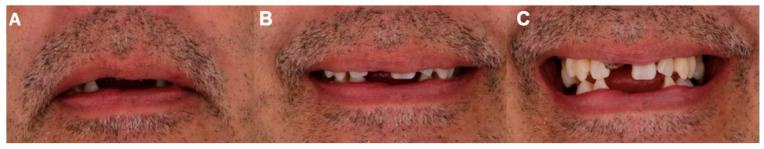
(**A**) Upper lip at rest. (**B**) Smile. (**C**) Maximum smile.

A dentolabial analysis was performed to determine the vertical dimension of occlusion (VDO) and to establish the incisal edge position. The patient had not experienced any loss of VDO, so the existing vertical dimension was maintained for the fabrication of the dentures. The incisal edge of tooth no. 21 was selected as a reference point to establish anterior guidance. At rest, the incisal edge exposure measured 2 mm (Figure 2).

Following this analysis, definitive impressions were taken using modeling compound for border molding (Kerr, Orange, CA, USA) and regular-consistency polyvinyl siloxane (Imprint II, 3M, St. Paul, MN, USA). The maxillary cast was mounted using a facebow transfer, while the mandibular cast was mounted in centric relation on a semi-adjustable articulator (Panadent, Colton, CA, USA), maintaining the vertical dimension of occlusion (VDO) as provided by the patient. No custom adjustments were made to the articulator and condylar adjustments were used at average manufacturer values since previous guidance of the remaining teeth was inadequate and tooth mobility made it difficult to take protrusive records for these modifications.

**Figure 3:**
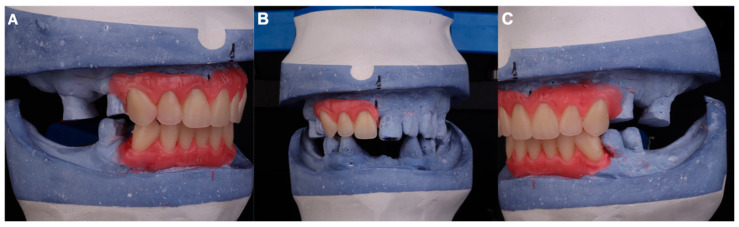
(**A**) Right lateral view showing elimination of upper occlusal interferences. (**B**) Wax mount of upper right incisors and canine. (**C**) Elimination of upper occlusal interferences on the left lateral view with 1 mm overjet and overbite.

A modification of the working model was carried out by removing teeth no. 13, 12, and 11 from the cast, using the incisal edge and midline provided by tooth no. 21 as reference points. Artificial teeth corresponding to teeth 13, 12, and 11 were positioned on the residual ridge using the technique described by Jerbi [16]. These newly placed teeth then served as the reference for removing teeth no. 21, 22, and 23 from the cast and replacing them with artificial teeth, thereby establishing the upper incisal edges [16]. The lower anterior sextant was subsequently removed from the cast, and artificial teeth from canine to canine were positioned, maintaining the midline and providing 1 mm of vertical overbite and 1 mm of overjet relative to the upper anterior incisal edges. Once the anterior guidance was determined, the establishment of the occlusal surfaces was initiated. Posterior interferences on the model were removed [16] to allow the proper positioning of the anteroposterior curve (Figure 3).

**Figure 4:**
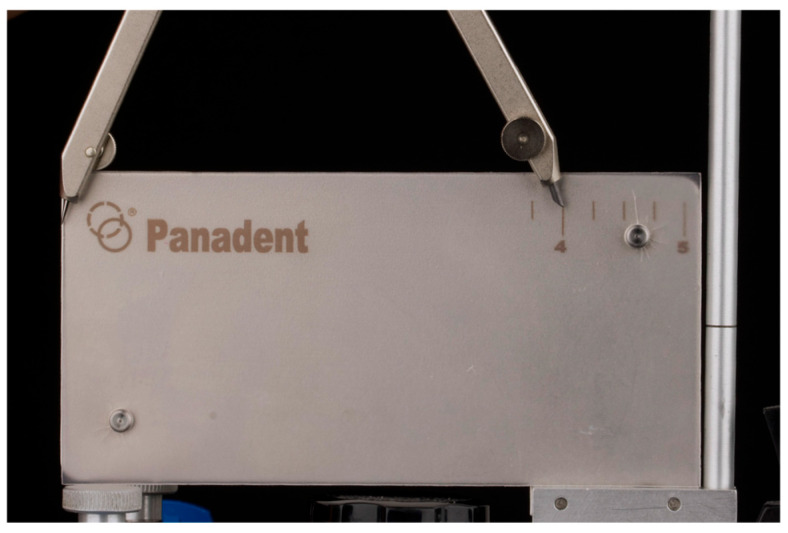
Selecting the 4-inch radius on the Broadrick flag with the compass.

Using the Broadrick Occlusal Plane Analyzer (BOPA) on the articulator and a compass, a radius of 4 inches (10.16 cm) was selected—an often-used measurement, particularly in patients with skeletal Class I relationships (Figure 4).

**Figure 5:**
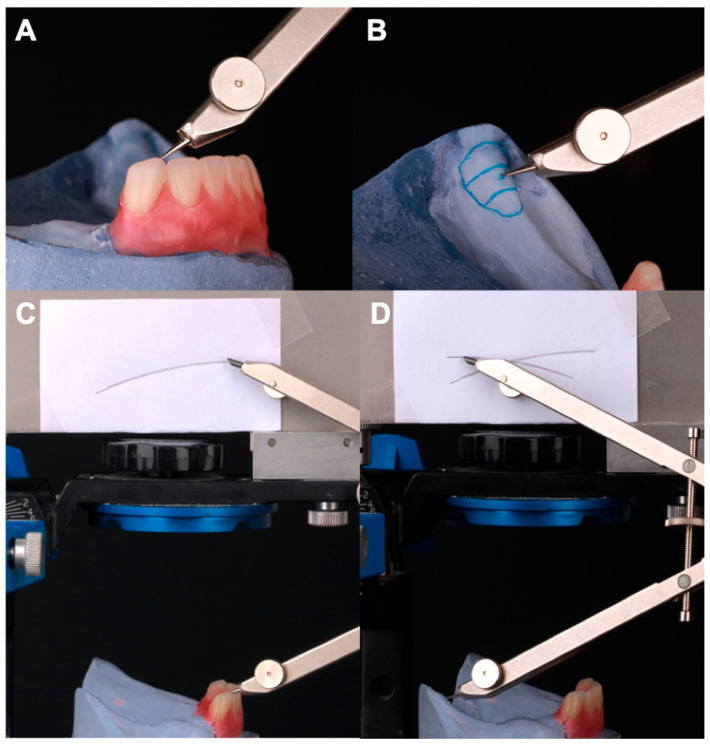
(**A**) The mandibular canine cusp corresponds to the Anterior Survey Point (ASP). (**B**) The middle third of the retromolar pad corresponds to the Posterior Survey Point (PSP). (**C**) The ASP tracing from the cusp of the lower canine. (**D**) PSP tracing from the middle third of the retromolar papilla.

The tracing on the flag card was created by placing the compass point, set to the predetermined radius (4 inches), on the Anterior Survey Point (ASP), which corresponded to the mandibular canine cusp. An arc was then drawn on the flag card using the graphite end of the compass. The following tracing was performed by placing the tip of the compass on the Posterior Survey Point (PSP). The middle third of the retromolar pad was used as the posterior reference point. Another arc was drawn on the flag card with the graphite end of the compass from the middle third of the retromolar pad; this arc intersected the initial arc drawn from the Anterior Survey Point (ASP), forming an intersection point between the ASP and PSP (Figure 5).

**Figure 6:**
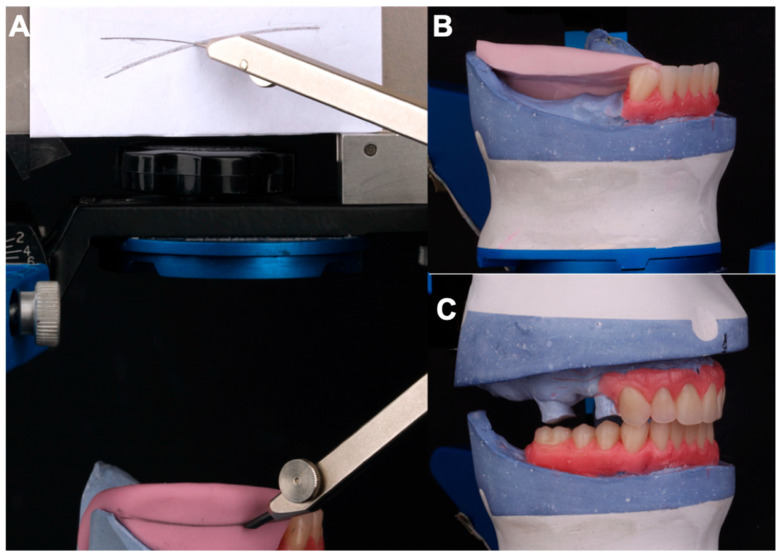
(**A**) Bisector point between the ASP and the PSP, with low curvature resulting from the traced over the putty material. (**B**) Cut putty with space for mounting teeth. (**C**) Posterior teeth mounted in wax.

Then, the tip of the compass was placed at the intersection point between the ASP and PSP to establish the reference point for the Center of the Occlusal Plane (COP). The occlusal plane was drawn on a heavy-body putty material (Zetalabor; Zhermack S.p.A., Badia Polesine, Italy) using the graphite end of the compass. This line served as a reference that was trimmed to clear the prosthetic space for the posterior occlusal plane and to allow the mounting of the posterior artificial teeth (Figure 6).

**Figure 7:**
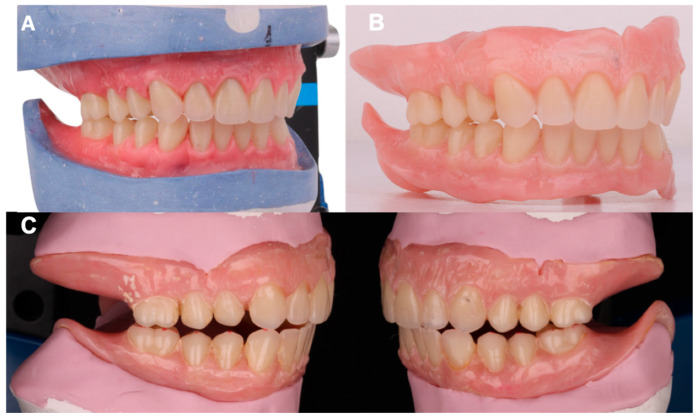
(**A**) Complete wax-up of artificial teeth with contacts in centric relationship. (**B**) Processed acrylic prosthesis. (**C**) Lateral contacts.

The remaining upper teeth were positioned in relation to the lower occlusal plane. Once all artificial teeth were placed in the prosthesis, occlusion was evaluated. Protrusive and lateral movements were performed on the semi-adjustable articulator, ensuring balanced posterior contacts during all functional movements, thereby achieving bilateral balanced occlusion. The dentures were processed with heat-curable acrylic (Opti-cryl; New Stetic S.A., Medellín, Colombia), cured for 8 h in a controlled-heating oven (Hanau, Germany), and occlusion was adjusted indirectly through laboratory remounting post-processing [17] (Figure 7).

**Figure 8:**
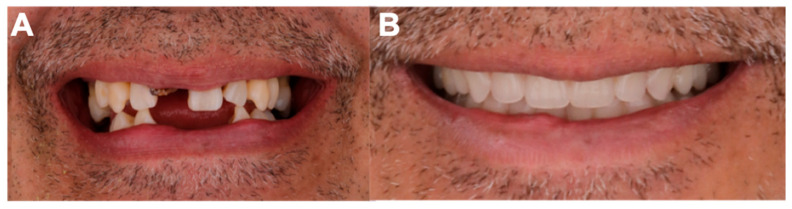
(**A**) Before oral rehabilitation; (**B**) After oral rehabilitation.

After the surgical procedure involving multiple extractions of all remaining teeth, the prostheses were delivered to the patient (Figure 8). A soft relining material (Flexacryl Soft; Lang Dental Mfg. Co., Inc., Wheeling, IL, USA) was applied inside the prosthesis to facilitate the adaptability during the healing process, finally, the patient was provided with instructions for use and hygiene, which include not removing dentures for the first 24 h to promote hemostasis.

Follow-up was performed after delivery at 72 h, after one week (once the sutures had been removed), after 15 days, and after one month. Over a period of 4 months, the internal surface of the prosthesis was adjusted in areas of excessive pressure using pressure-indicating paste (Mizzy PIP; Keystone Industries, Gibbstown, NJ, USA) and sites indicated by the patient as causing discomfort. Additionally, some soft internal relines were applied to compensate for the bone remodeling process and improve patient comfort during the healing process. Definitive complete dentures will be fabricated once the alveolar process healing is stable.

## 3. Discussion

Accurate determination of the occlusal plane is an important factor in the design of immediate complete dentures, as it supports normal function of the muscles, tongue, and cheeks, thereby enhancing prosthesis stability. Ideally, the occlusal plane should replicate the position of the patient’s natural dentition [10]. It should be noted that it is not a plane as such, but rather represents the planar average of the curvature of the occlusal surfaces [10]. The plane of occlusion reflects the average curvature of the occlusal surface, defined by the incisal and occlusal surfaces of the teeth—commonly referred to as the curve of Spee [18,19]. Graf von Spee located the center of this curve along “a horizontal line through the middle of the orbits behind the posterior crista lachryma,” a concept later expanded by George Monson, who proposed that the anteroposterior curve is part of a three-dimensional sphere with its center of rotation near the glabella, and a radius of approximately 4 inches (10.16 cm), particularly in Class I skeletal relationship, or depending on the skeletal class, radius of 3 ¾ inches (9.54 cm) for Class II skeletal relationship and a radius of 5 inches (12.17 cm) for Class III skeletal relationship [18,19,20]. Monson’s curve integrates both the curves of Spee and Wilson into a three-dimensional occlusal pattern [11].

Several methods exist for establishing an acceptable occlusal plane, including direct analysis via selective grinding of natural teeth, indirect analysis using facebow-mounted casts with properly adjusted condylar paths, and the Broadrick Occlusal Plane Analyzer (BOPA). When restoration of most or all posterior teeth is required, the BOPA technique offers a straightforward preliminary method for locating cusp positions on the diagnostic casts [19].

Lawson developed a flag-shaped instrument that attaches to the upper horizontal arm of various articulators, allowing with the aid of a compass, two intersecting arcs to be drawn with a radius of 4, 3 ¾, or 5 inches, depending on the skeletal relationship of each patient. The intersection point of these curves will guide the location of the center of the Occlusal Plane (COP) and optimize the posterior occlusal orientation in harmony with anterior and condylar guidance [11,19]. The mandibular canine cusp serves as the Anterior Survey Point (ASP), while the distobuccal cusp of the most distal molar, or, in its absence, some authors have recommended using a portion of the retromolar pad as the Posterior Survey Point (PSP). In edentulous patients, the PSP is usually determined by the approximate junction of the upper or middle third of the retromolar pad and the ASP [10,21,22].

The Broadrick flag identifies the most probable center of the curve of Spee; however, it should be considered that this position is neither fixed nor unalterable. Esthetic and functional demands may require adjustments to the center, which should always lie along the arc drawn from the ASP, but may be shifted anteriorly or posteriorly from its intersection with the PSP arc. This displacement is possible by selecting the upper third, the middle third, or the junction point of these as the PSP at convenience. The retromolar pad is a pear-shaped area that forms only after the extraction of the most distal molar. It is a soft tissue region and normally coincides with the occlusal plane of the mandibular arch. Occasionally, its anterior and posterior borders cannot be precisely delineated, and its inclination and anatomy vary among patients [10,23]. An anterior displacement of the PSP deepens the curve, while a posterior displacement flattens it. An anterior shift in PSP steepens the curve, while a posterior shift flattens it [11,12,20]. A flatter curve may introduce posterior protrusive interferences due to a mismatch between the curve of Spee and the condylar guidance angle [11,12]. These posterior interferences can facilitate the establishment of a bilateral balanced occlusal scheme during the fabrication of a complete denture. This scheme promotes the stability and retention of the prosthesis by maintaining occlusal contacts in centric relation during eccentric, lateral, and protrusive mandibular movements [7,24,25].

This clinical case exemplifies the application of the classic Lawson Broadrick technique to establish the posterior occlusal plane, facilitated by the presence of remaining upper anterior incisors with intact incisal edges, which were used as a reference to develop the new anterior guidance with the artificial teeth. This article describes a unique clinical case; therefore, the results are not generalizable and depend on anatomical variations. However, the detailed analysis of the procedure and clinical decisions provides valuable information for analysis and learning in similar contexts.

## 4. Conclusions

During the design of an immediate complete denture, both esthetic and functional factors must be carefully considered to provide patient comfort. One of the main challenges lies in establishing a new occlusal plane, especially when the remaining teeth scheduled for extraction restrain the clinical try-in of the artificial teeth. This clinical report presents a qualitative clinical case study of a single patient, illustrating the use of the Broadrick Occlusal Plane Analyzer, which facilitated the establishment of an occlusal plane in harmony with the anterior and condylar guidance. Patient comfort was determined through the patient’s personal opinion, who used them temporarily during the healing process.

## Data Availability

The original data presented in the study are included in the article, further inquiries can be directed to the corresponding author.
